# 

**Published:** 1896-12

**Authors:** 


### Newport Medical Society.

W. Basset, *Hon. Sec.*

### The Library of the Bristol Medico-Chirurgical Society.

At the Annual Meeting, held on October 14th, 1896, Mr. L. M. Griffiths, the Honorary Librarian, read the following Report:—

Since the last Annual Meeting 686 volumes have been received into the Society's library. As 43 volumes have been given to other libraries, the number of volumes actually in the library at the present time, including 556 duplicates, is 5173. The periodicals reserved by the Society number 158. Many volumes of periodicals are required to complete sets. Gifts of these, or money for the purpose of obtaining them, will be very acceptable.

The donors of books during the twelve months have been:— The Bootle Public Library, Mr. C. W. J. Brasher, the British Medical Association, Dr. J. Michell Clarke, Mr. F. R. Cross,

Dr. George M. Gould, Mr. L. M. Griffiths, l'Institut international de Bibliographie, Mr. C. M. Jessop, the Laryngological Society of London, the Ophthalmological Society of the United Kingdom, Mr. Stephen Paget, Mr. E. Picton Phillips, Dr. W. Warren Potter, the Royal Academy of Medicine in Ireland, the Royal College of Surgeons of England, Mrs. R. B. Ruddock, Mr. E. J. Sheppard, Mr. J. Greig Smith, Dr. R. Shingleton Smith, the Surgeon-General United States Army, Dr. James Swain, Dr. P. Watson Williams, and Messrs. J. Wright & Co. One framed picture has been presented by Mr. G. Barry Hart and one by Mr. H. E. Hetling.

A library such as ours should, in addition to containing the best medical books and periodicals of to-day, aim at collecting the literature of bygone ages, thus preserving for us in its original form the excellent work done by our forefathers in building up the present noble structure of medical art and practice. In the library also should be found a place for matters of a more personal and local nature, which in the future may prove of interest, if not of use, to the gleaner of half-forgotten trifles. These would include newspaper cuttings, pamphlets and magazine articles of ephemeral value, professional reports, portraits, illustrations of things or places having a medical bearing, and many other items which will occur to every member. Any of these will at all times be gladly received.

I feel sure that as time goes on the members of this Society appreciate more and more the peculiar constitution of the Bristol Medical Library,[1] which, uniting four libraries of the chief Medical Institutions of the city, now enables them to consult a collection of 15,362 volumes and 194 current periodicals.

---

*The following donations have been received since the publication of the list in September:*

*November 30th, 1896.*

| | |
|---|---|
| J. Michell Clarke, M.D. (1) | 3 volumes. |
| J. Ewens (2) | 1 volume. |
| L. M. Griffiths (3) | 5 volumes. |
| W. H. Harsant (4) | 1 volume. |
| W. Warren Potter, M.D. (5) | 3 volumes. |
| Lady Reynolds (6) | 157 ,, |
| J. Greig Smith (7) | 1 volume. |
| R. Shingleton Smith, M.D. (8) | 13 volumes. |

[1] By the courtesy of the authorities of University College I am able to give a reproduction of the latest photograph of the library that has been taken. The picture, however, does not show a third of the actual shelf accommodation.

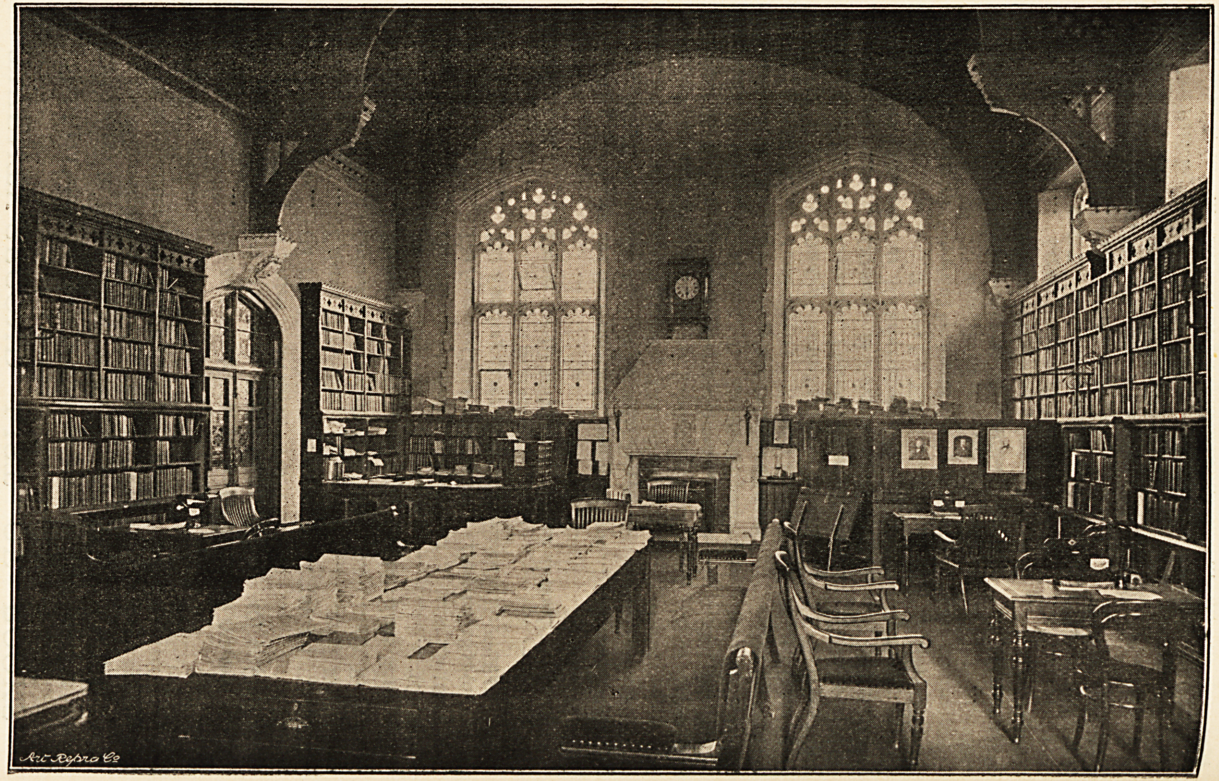

MEDICAL LIBRARY, UNIVERSITY COLLEGE, BRISTOL.

### TWENTY-SECOND LIST OF BOOKS.

The titles of books mentioned in previous lists are not repeated.

The figures in brackets refer to the names of the donors, and show by whom the volumes were presented. The books to which no such figures are attached have either been bought from the Library Fund or received through the *Journal*.

**Abercrombie, J.** ... *Des Maladies de l'Encéphale et de la Moelle épinière.* (Tr. par A. N. Gendrin). ... ... (6) 2e Éd. 1835
**Allbutt, T. C.** [Ed.] *A System of Medicine.* Vol. I ... ... ... ... ... 1896
**Althaus, J.** ... ... *On Paralysis, Neuralgia, &c.* ... ... (6) 3rd Ed. 1864
**Barr, T.** ... ... ... *Diseases of the Ear* ... .. ... ... ... 2nd Ed. 1896
**Barwell, R.** ... ... *Lateral Curvature of the Spine* ... ... ... 5th Ed. 1895
**Béclard, P.-A.** ... *Elémens d'Anatomie générale* ... (6) Nouv. Éd. 1828
**Beigel, H.** ... ... *On Inhalation* ... ... ... ... ... ... ... ... (6) 1866
**Billing, A.** ... ... *On Diseases of the Lungs and Heart* ... ... ... (6) 1852
**Bowles, R. L.**... ... *The Inaugural Address, St. George's Hospital,* 1892 (6) 1892
**Bruck, L.** ... ... *The Sweating of the Medical Profession by the Friendly Societies in Australasia* ... ... ... ... ... ... [1896]
**Charcot, J.-M.** ... *Leçons sur les Maladies du Système nerveux.* 2 vols. (8) 3e Éd. 1877
*Chloroform, The Lancet and the Hyderabad Commissions on* ... ... ... ... [N.D.]
**Clarke, W. M.** ... *The Natural History of Disease* ... ... ... ... (3) 1879
*Climates and Baths of Great Britain, The.* Vol. I. ... ... ... ... ... 1895
*Clubs, The Battle of the* .. ... ... ... ... ... ... ... ... ... ... [1895-96]
**Colman, W. S.** ... *Section Cutting and Staining* ... ... ... 2nd Ed. 1896
**Cornil et V. Hanot, H. Hérard, V.** *La Phtisie pulmonaire* (8) 2e Éd. 1888
**Crookshank, E. M.** *Bacteriology and Infective Diseases* ... ... 4th Ed. 1896
**Dalby, Sir W. B.** ... *Aural Surgery* ... ... ... ... ... ... ... 3rd Ed. 1896
**Davey, J. G.** ... ... *On Life and its Lessons* ... ... ... ... ... ... (3) 1883
**Davy, J.** ... ... ... *Researches, Physiological and Anatomical.* 2 vols. (6) 1839
**Debove, —** ... ... *Leçons sur la Tuberculose parasitaire* ... ... .. (8) 1884
*Dictionnaire (Nouveau) de Médecine et de Chirurgie pratiques* (Jaccoud). Tomes I.—XIII. .. ... ... ... ... ... (6) 1864-70
*Diphtheria Antitoxic Serums, The Lancet Special Commission on the relative strengths of* ... ... ... ... ... ... ... ... ... 1896
**Doyen, E.** ... ... *Traitement des Suppurations pelviennes* ... ... ... 1896
**Ewart, W.** ... ... *Gout and Goutiness* ... ... ... ... ... ... ... ... 1896
**Falret, [J.-P.]** ... *Leçons cliniques de Médecine mentale* ... ... ... (6) 1854
**Farquhar, T. Fox and T.** *Endemic Skin and other Diseases of India* (8) 1876
**Fox, E. L.** ... ... *The Medulla Oblongata* ... ... ... ... ... ... (3) 1881
**Fox and T. Farquhar, T.** *Endemic Skin and other Diseases of India* (8) 1876
**Foxwell, A.** ... ... *The Enlarged Cirrhotic Liver* ... ... ... ... ... [1896]
*Glasgow Pathological and Clinical Society—Discussion on Albuminuria* (8) 1884
**Goodman, J.** ... ... *Nervous Origin of Disease* ... ... ... ... ... ... (6) 1854
**Gould, G. M.**... ... *The Student's Medical Dictionary* ... ... 10th Ed. 1896
**Green, J. H.** ... ... *Mental Dynamics* ... ... ... ... ... ... ... ... (6) 1847

**Haig-Brown, Florence A.** *Hints on Elementary Physiology* ... ... ... 1896
**Halliburton, W. D.** *Kirkes' Hand-Book of Physiology* ... ...14th Ed. 1896
**Hanot, H. Hérard, V. Cornil et V.** *La Phtisie pulmonaire* ... (8) 2e Éd. 1888
**Hartridge, G.** ... ... *Refraction of the Eye* ... ... ... ... ... 8th Ed. 1896
*Harvard University, Addresses and Exercises at the One Hundredth Anniversary of the Foundation of the Medical School of, Oct. 17th,* 1883 ... ... ... ... ... ... (6) 1884
**Heberden, W.** ... *Commentarii de Morborum Historia et Curatione* (6) Editio Altera 1807
**Hérard, V. Cornil et V. Hanot, H.** *La Phtisie pulmonaire* (8) 2e Éd. 1888
**Hewitt, G.** ... ... *The Diseases of Women* ... (6) 2nd Ed., 1868; (6) 3rd Ed. 1872
**Hoblyn, R. D.** ... *Dictionary of Medical Terms* ... ... (6) 9th Ed. 1868
**Holth, S.** ... ... ... *Synsfeltundersogelser* ... ... ... ... ... ... ... ... 1896
*Infectious Disease (Notification) Act, Difficulties under the* ... ... ... .. 1895
**Jackson, E.** ... ... *Skiascopy* ... ... ... ... ... ... ... 2nd Ed. 1896
**James, A.** ... ... *Cliniques* ... ... ... ... ... ... ... ... ... ... 1896
**Kirkes, [W. S.]** ... *Hand-Book of Physiology.* 14th Ed. By W. D. Halliburton ... ... ... ... ... ... ... ... 1896
**Lee, A. B.** ... ... *The Microtomist's Vade-Mecum* ... ... ... 4th Ed. 1896
**Mahlendorff, P.** ... *Voice Production* ... ... ... ... ... ... ... ... ... [N.D.]
**Manners-Smith, B. C. A. Windle and T.** *Surface Anatomy* ... 2nd Ed. 1896
**Meryon, E.** ... ... *On Paralysis* ... ... ... ... ... ... ... ... ... (6) 1864
**Miquel et A. Rueff, P.** *Tuberculose pulmonaire* ... ... ... ... ... (8) 1888
*Pathological Society of London, Catalogue of the Preparations, Drawings, etc., exhibited at the Jubilee of the* ... ... ... (8) 1896
**Peacock, T. B.** ... *Valvular Disease of the Heart* ... ... ... ... (6) 1865
*Physiology, An American Text-Book of* (Ed. by W. H. Howell) ... ... 1896
**Powell, G. D.** ... *Medical Electricity* ... ... ... ... ... ... ... (6) 1869
*Plumbers' Work, The Lancet Special Commission on* ... ... ... ... ... ... 1896
**Richards, P. A. E.** *Practical Chemistry* ... ... ... ... ... ... ... ... 1896
**Ridge, B.** ... ... *Physiology of the Uterus, Placenta, and Fœtus* ... (6) 1845
,, ... ... *Glossology* ... ... ... ... ... ... ... (6) 2nd Ed. 1857
**Rostan, L.** ... ... *Recherches sur le Ramollissement du Cerveau* (6) 2e Éd. 1823
**Rueff, P. Miquel et A.** *Tuberculose pulmonaire* ... ... ... ... ... ... (8) 1888
**Saundby, R.** ... ... *Lectures on Renal and Urinary Diseases* ... 2nd Ed. 1896
**Semb, O.** ... ... ... *Om de papillære Ovarial-kystomer* ... ... ... ... 1896
**Simson, T.** ... ... *On Vital and Animal Actions* ... ... ... ... (6) 1752
**Spallanzani, [L.]** ... *Tracts on the Natural History of Animals and Vegetables.* 2 vols. (Tr. by J. G. Dalyell) (6) 2nd Ed. 1803
**Swayne, J. G.** ... *Medical Diagnosis and Treatment, Past and Present* (3) 1880
**Swieten, G. van** ... *Commentaries upon the Aphorisms of Boërhaave.* Translated. (6) Vols. II.—V., 1754-59; VII., VIII., 1758; X.—XIV., 1765
*Therapeutics, An American Text-Book of Applied* (Ed. by J. C. Wilson and A. A. Eshner.) 2 vols. ... ... ... ... ... 1896
**Tilt, E. J.** ... ... *On Uterine Therapeutics* ... ... ... (6) 3rd Ed. 1868
**Tubby, A. H.** ... ... *Deformities* ... ... ... ... ... ... ... ... ... 1896

*Tuberculose, Études sur la.* Tomes I.; II., fasc. i. ... .. ... (8) 1887-88

**Tweedie, A.** [Ed.] ... *A System of Practical Medicine.* 5 vols.... ... (6) 1840

**Webster, J. C.** ... *Practical and Operative Gynecology* ... ... ... ... 1896

**West, C.** ... ... ... *The Profession of Medicine...* ... ... ... ... .. 1896

**Windle and T. Manners-Smith, B. C. A.** *Surface Anatomy* ... 2nd Ed. 1896

---

### TRANSACTIONS, REPORTS, JOURNALS, &c.

Académie royale de Médecine, Mémoires de l' (6) Tomes V.—XVI. 1836-52
Alabama, Transactions of the Medical Association of the State of ... 1896
American Association of Obstetricians and Gynecologists, Transactions of the ... ... ... ... ... ... ... ... ... ... ... Vol. VIII. 1896
Annals of Surgery ... ... ... ... Vols. (4) IX., 1889; XIII., XIV. 1891
Archives de Neurologie ... ... ... ... (1) Tomes XVII.—XXII. 1889-91
British Balneological and Climatological Society, Transactions of the 1896
British Medical Association, Memorandum and Articles of Association and By-laws of the ... ... .. ... ... ... ... ... .. ... (3) 1883
British Orthopædic Society, Transactions of the ... ... (2) Vol. I. 1896
Buffalo Medical and Surgical Journal ... ... Vols. (7) XXIX., 1890; (5) XXX.—XXXII. 1891-93
Clinical Journal, The ... ... ... ... ... ... ... ... Vol. VIII. 1896
Clinical Society's Transactions ... ... ... ... ... ... Vol. XXIX. 1896
Congrès pour l'Étude de la Tuberculose, 1e Session, 1888. 2 vols. (8) 1889
Dermatological Society of Great Britain and Ireland, Transactions of the ... ... ... ... ... ... ... ... ... ... ... ... ... ... Vol. II. 1896
Epidemiological Society of London, Transactions of the. N.S. Vol. XV. 1896
Hospital, The ... ... ... ... ... ... ... ... ... ... Vol. XX. 1896
Medical Chronicle, The ... ... ... ... ... ... ... ... N.S. Vol. V. 1896
Medical News, The ... ... ... ... ... ... ... ... Vol. LXVIII. 1896
Medical Times and Gazette, The (6) 1876, Vol. II.—1877, Vol. II., 1879-80
Michigan State Medical Society, Transactions of the ... Vol. XX. 1896
New Hampshire Medical Society, Transactions of the ... ... ... ... 1896
Ohio State Medical Society, Transactions of the ... ... ... ... ... 1896
Philosophical Transactions, Abstracts of the, 1800-30. 2 vols. (6) 1832-33
Practitioner, The ... ... ... ... ... ... ... ... ... ... Vol. LVI. 1896
Quarterly Medical Journal, The ... ... ... ... ... ... ... ... Vol. IV. 1896
South Carolina Medical Association, Transactions of the ... ... ... 1896
University Medical Magazine ... ... ... ... ... ... ... Vol. VIII. 1896
University of London—Minutes of the Senate ... ... ... ... ... (6) 1881
West London Medico-Chirurgical Society, Proceedings of the. Vol. VII. 1896

---

### Local Medical Notes.

### BRISTOL.

At a quarterly meeting of the Comitia of the Royal College of Physicians of London, held on October 29th, Theodore Fisher, M.D. Lond., was elected a Member.

University College.—The Annual Distribution of Prizes to the successful students of the Faculty of Medicine took place in the Medical Library of the College on November 25th. Mr. Albert Fry, Chairman of the Council, presided, and in his address to the students said that he occupied his present position at the request of the Faculty of

Medicine. The Council were extremely anxious that the medical department, now that it was housed in such complete and handsome buildings, should continue worthy of its old reputation, and should also make full use of its present advantages. Every facility was afforded for the acquirement of a complete and thorough education, and students could not too clearly bear in mind that now was the time when the foundation of their future life would be laid. It should be their aim by their diligence to make the College one of the most eminent schools of medicine in the United Kingdom, and in advancing its reputation they would do honour to themselves and promote their own success in after life.

The following students have recently passed examinations:

**University of London.**—*Final Examination for M.B. Degree* (Second Division)—G. B. Price, M.R.C.S. Eng., L.R.C.P. Lond. (1st Class Honours in Obstetric Medicine).

**University of Durham.**—*Examination for M.B. Degree. First and Second Examinations*—C. O. Bodman.

**Examining Board in England by the Royal Colleges of Physicians and Surgeons.** — *Four Years' Curriculum. First Examination. Part II. Materia Medica* — F. C. Morgan, J. W. Wallace. *Five Years' Curriculum. First Examination. Part III. Elementary Biology* — F. H. Rudge. *Second Examination. Anatomy and Physiology*—H. Hemsted, P. G. Stock. *Final Examination. Medicine* — F. Anthony. *Surgery* — T. W. W. Bovey,* W. H. Cooper,* E. V. R. Woodford,* W. Smith.* *Midwifery*—F. P. Gill, J. J. S. Lucas, F. P. Mackie, J. C. MacWatters.*

**Royal College of Surgeons of England.** — *Fellowship. First Examination*—R. H. Chilton, M.R.C.S., L.R.C.P.Lond. *Final Examination* — A. E. H. Pinch, M.R.C.S., L.R.C.P. Lond.; W. M. Willis, M.R.C.S., L.R.C.P. Lond.

J. Odery Symes, M.D. Lond., has been appointed a Demonstrator of Anatomy.

HOSPITAL FOR SICK CHILDREN AND WOMEN.—E. C. Taylor, M.D. Lond., F.R.C.S. Eng., has been elected House-Surgeon.

### EXETER.

DEVON AND EXETER HOSPITAL.—Rapid progress is being made with the new wing to this hospital. The total length of the new wing, including the sanitary towers, is about 140 feet, and from the ground to the ridge the average height is over 60 feet. The main walls are of brickwork, 18 in. in thickness throughout. The wing is three storeys high, and consists of three main wards, each containing twenty-four beds, and three small single-bed wards. The three main wards are 97 feet long, 26 feet 6 in. wide; each will be warmed by means of two central stoves (the vertical flues of which will be swept from the ground level) and eight hot-water coils. Each patient has 1,500 cubic feet of air space in the main wards. The floors are fire-proof, and are formed of steel joists with concrete and terra-cotta lintels, on which are laid teak floor-boards. At the further end of the wing there is a balcony to each floor, and a fire-escape staircase. The space underneath the wards will form a covered airing ground. Each floor has a linen-room, kitchen, pantry, etc.; and in the towers are bath-rooms, lavatories, etc., the walls of which are lined with white glazed

* Completes Examination.

bricks inside. An electric lift will be provided, running from the basement to the top floor, for patients, etc. The whole of the building is to be lit with the electric light, and there will be twelve wall plugs in each ward. Under the new arrangement the kitchens will occupy a floor of the old central building, and provision is to be made by a new system of lifts for the rapid distribution of meals to the various wards. Mr. C. Cole, 50 High Street, Exeter, is the architect.

---

**Alvarenga Prize of the College of Physicians of Philadelphia.**—The next award of this prize will be in July, 1897. Essays intended for competition may be upon any subject in Medicine. Particulars may be obtained from the Secretary of the College.

---

### SCRAPS

### LIST OF SUBSCRIBERS

TO

### The Bristol Medico-Chirurgical Journal.

*DECEMBER*, 1896.

---

### England.

### CAMBRIDGESHIRE.

CHATTERIS.
C. J. A. Coates

### CHESHIRE.

MACCLESFIELD.
W. R. Etches

NORTHWICH.
P. T. Tolputt

### CORNWALL.

EAST LOOE.
L. F. Houghton

FALMOUTH.
S. A. Lidiard

NEW QUAY.
A. Hardwick

PENZANCE.
W. S. Bennett
J. A. Fox
J. Symons

ROCHE.
W. R. Goodfellow

ST. AUSTELL.
W. Mason

### DEVONSHIRE.

BABBACOMBE.
T. Finch

BARNSTAPLE.
C. H. Gamble
J. Harper
M. Jackson

BRIXHAM.
G. C. Searle

BROAD CLYST.
J. Somer

BUDLEIGH SALTERTON.
T. G. C. Evans
H. F. Semple

CHAGFORD.
A. D. Hunt

CHUDLEIGH.
C. L. Cunningham

COLYTON.
T. L. Callaghan.

CULLOMPTON.
G. G. Gidley

DARTMOUTH.
R. B. Searle

DAWLISH.
F. H. Hudson

DEVONPORT.
F. E. Row

EXETER.
J. M. Ackland
A. G. Blomfield
H. Davy
G. T. Clapp
E. J. Domville
C. Fenwick
A. W. Kempe
J. MacKeith
R. V. Solly
E. B. Steele-Perkins
L. H. Tosswill

HONITON.

T. W. Shortridge

ILFRACOMBE.
F. W. Langridge

MILLBROOK.
A. B. Cheves

MORTHOE.
E. J. Hollings

NEWTON ABBOT.
J. Culross

PAIGNTON.
J. Alexander

PLYMOUTH.
G. F. Aldous
R. H. Clay
R. Lucy
Medical Society
E. B. Thomson
W. L. Woollcombe

### DEVONSHIRE *(Continued).*

PLYMPTON.

C. Aldridge
W. D. Stamp

SIDMOUTH.

J. H. S. Pullin

TEIGNMOUTH.

G. H. Johnson

TOPSHAM.

J. N. Frood

TORQUAY.

H. Humphreys
W. J. K. Millard
W. Odell
R Pollard
W. Powell
Medical Society
J. T. Thomas
C. H. Wade

UFFCULME.

F. Morgan

WILLAND.

H. E. Tracey

WINKLEIGH.

J. H. Norman

### DORSETSHIRE.

BEAMINSTER

T. P. Daniel

DORCHESTER.

P. W. MacDonald

POOLE.

J. McNicoll

PORTLAND.

G. H. Lilley

STURMINSTER NEWTON.

J. C. Leach

WIMBORNE.

A. J. H. Cresp

WEYMOUTH.

J. M. Lawrie

### ESSEX.

NEWPORT.

W. A. Smith

### GLOUCESTERSHIRE.

BRISTOL.

W. R. Ackland
G. Adams
J. Ambrose
C. Atchley
W. M. Barclay
T. G. L. Baretti
B. J. Baron
A. E. Blacker
A. F. Blagg
E. C. Board
C. W. J. Brasher
J. Broom
C. A. La T. Brough
F. St. J. Bullen
E. F. H. Burroughs
J. P. Bush
A. Carr
T. M. Carter
W. F. Carter
T. Carwardine
R. H. Chilton
J. M. Clarke
T. V. Coker
H. Cook
W. H. Cory
W. Cotton
F. R. Cross
J. Dacre
D. S. Davies
H. F. Devis
N. C. Dobson
W. Dowson
E. L. W. Dunbar
E. Eberle
F. H. Edgeworth
W. Elder
C. Elliott
J. Ewens
E. Fawcett
R. G. Fendick
J. L. Firth
T. Fisher
E. L. Fox
J. Freeman
J. Fuller
W. J. Fyffe
E. B. Garland
T. T. Genge
D. S. Gerrish
A. N. G. Gibbs
G. A. Gloag
W. G. Grace
J. S. Griffiths
L. M. Griffiths
C. D. G. Hailes
A. H. Haines
A. J. Harrison
W. H. Harsant
A. G. Hayman
C. A. Hayman
J. C. Heaven
C. K. C. Herapath
H. E. Hetling
H. Hill
T. Hill
General Hospital
G. A. Imlay
T. B. Kelly
H. W. Kendall
F. P. Lansdown
R. G. P. Lansdown
A. E. A. Lawrence
E. L. Lees
W. Ligertwood
P. W. McCrea
H. Marshall
R. Martin
C. E. Matthews
T. O. Mayor
H. F. Mole
C. A. Morton
G. T. Myles
B. G. Neale
W. N. Nevill
C. Newman
W. H. C. Newnham
T. D. Nicholson
J. A. Norton
C. O'Brien
A. Ogilvy
G. S. Page
G. Parker
J. H. Parry
A. W. Peake
W. A. Perry
C. F. Pickering
A. E. H. Pinch
H. I. Pocock
W. E. Pountney
J. J. Powell
A. Prichard
A. W. Prichard
J. E. Prichard
A. Pring
A. B. Prowse
M. Randall
W. Richardson
B. M. H. Rogers
W. B. Roué
C. K. Rudge
H. T. Rudge
J. E. Shaw
E. J. Sheppard
E. M. Skerritt

### GLOUCESTERSHIRE *(Continued)*.

BRISTOL *(continued)*.

G M. Smith
J. G. Smith
R. S. Smith
C. Steele
J. B. Stephenson
W. H. Stevens
F. W. Stoddart
J. Swain
J. G. Swayne
S. H. Swayne
W. C. Swayne
J. O. Symes
E. C. Taylor
J. Taylor
W. J. Tivy
H. Waldo
C. H. Walker
C. H. Wallace
J. H. Wathen
J. Wilding
C. Williams
P. W. Williams
H. W. Windsor-Aubrey
C. Wintle

CHELTENHAM.

C. E. F. Mouat-Biggs
G. A. Peake
E. Trevithick
L. Winterbotham

CHIPPING SODBURY.

A. Grace

CINDERFORD.

W. C. Heane

CIRENCESTER.

W. R. Cossham
O. H. Fowler
C. Mackinnon

COLEFORD.

L. B. Trotter

DOWNEND.

H. Skelton

FISHPONDS.

W. Brown

GLOUCESTER.

R. W. Batten
A. H. Benson
J. G. Soutar

HAMBROOK.

E. Crossman
F. W. Crossman
W. F. B. Eadon

KINGSWOOD.

R. W. Brimacombe

LYDNEY.

W. P. Kennedy

MINCHINHAMPTON.

B. Church

MORETON-IN-MARSH.

L. K. Yelf

PAINSWICK.

W. B. Fergusson

NAILSWORTH.

C. J. Power

PARKEND.

W. C. Halpin

REDFIELD.

J. W. Rodgers

STAPLE HILL.

W. Murray

STAPLETON.

H. T. S. Aveline
H. A. Benham

STOKE BISHOP.

H. A. Beaver
C. E. Wedmore

STONEHOUSE.

G. T. B. Watters

STOW-ON-THE-WOLD.

E. Dening

STROUD.

A. S. Cooke

E. A. Howsin

H. R. MacDougal.

TETBURY.

W. Wickham

THORNBURY.

E. M. Grace
T. H. Taylor
L. H. Williams

WESTBURY-ON-TRYM.

A. Harvey
H. Ormerod
H. L. Ormerod

WINTERBOURNE.

R. Eager

WOTTON-UNDER-EDGE.

T. C. Boyce
D. H. Forty

### HAMPSHIRE.

ALDERSHOT.

J. C. Barr

ALTON.

W. L. P. Bevan

BOURNEMOUTH.

J. S. Dickie
D. C. Embleton
J. G. Harsant
J. Horsfall

CARISBROOKE.

J. Groves

DROXFORD.

A. H. Goodwyn

FLEET.

H. Wilcox

HURSTBOURNE TENNANT.

A. W. Riddell

LYMINGTON.

J. Rendall

SOUTHAMPTON.

W. R. Y. Ives

SOUTHSEA.

J. W. Cousins

VENTNOR.

E. R. Woodford

### HERTFORDSHIRE.

ST. ALBAN'S.

A. H. Boys

### HUNTINGDONSHIRE.

ST. NEOT'S.

T. C. Grey
J. H. Hemming

### LANCASHIRE.

LIVERPOOL.

G. A. Hawkins-Ambler
Medical Institution
C. Williams

POULTON-LE-FYLDE.

P. H. Day

PRESTON.

J. E. Garner

### MIDDLESEX.

### LONDON.

J. Berry
J. F. Evans
T. C. Fox
M. Handfield-Jones
W. H. A. Jacobson
H. M. Jones
J. Lister
E. J. Maclean
J. Macready
A. L. Marshall
F. T. Roberts
W. J. Seward
F. J. Wethered
W. M. Willis
G. S. Woodhead

### TOTTENHAM.

W. H. Plaister

### MONMOUTHSHIRE.

### BLAENAVON.

A. B. Avarne.

### NEWPORT.

W. Basset
C. B. Gratte
H. R. Hudson
O. E. B. Marsh
A. G. Thomas
H. E. Williams

### PONTYPOOL.

S. B. Mason

### RHYMNEY.

T. H. Redwood

### RISCA.

G. B. Robathan

### TREDEGAR.

G. A. Brown

### NORFOLK.

### WALPOLE ST. ANDREWS.

R. Smith

### NOTTINGHAMSHIRE.

### NOTTINGHAM.

C. B. Taylor

### OXFORDSHIRE.

### HENLEY-ON-THAMES.

J. Lidderdale

### OXFORD.

R W. Doyne
H. P. Symonds

### SOMERSETSHIRE

### BATH.

G. A. Bannatyne
C. S. W. Cobbold
T. Cole
F. S. Cowan
S. Craddock
W. M. Ellis
A. E. W. Fox
H. W. Freeman
H. F. A. Goodridge
T. B. Goss
P. King
F. Lace
T. P. Lowe
R. J. H. Scott
W. H. Symons
L. H. Walsh
L. A. Weatherly
J. Wigmore

### BECKINGTON.

W. G. Evans

### BLAGDON.

T. O. Halliwell

### BRIDGWATER.

F. J. C. Parsons
R. H. F. Routh
J. B. Sincock
W. C. Thompson

### BRISLINGTON.

B. B. Fox
G. E. Crallan

### BURNHAM.

F. C. Berry

### CASTLE CARY.

C. Coombs

### CHEDDAR.

E. H. Openshaw
R. W. Statham

### CHEW MAGNA.

E. T. Hale
R. H. Brew

### CHILTON POLDEN

G. Campbell

### CLEVEDON.

H. J. Capron
T. Davis
W. J. Hill

### CREWKERNE.

E. J. Cave
W. J. Penny
W. W. Webber

### CURRY RIVEL.

R. H. Vereker

### FRESHFORD.

C. E. S. Flemming

### FROME.

A. W. Dalby
J. M. Rattray

### HIGHBRIDGE.

W. R. Hadwen

### ILMINSTER.

C. Munden

### KEYNSHAM.

C. Harrison
G. G. D. Willett

### SOMERSETSHIRE *(Continued).*

LANGPORT.
J. Morgan

LIMPLEY STOKE.

C. J. Whitby

MIDSOMER NORTON.
A. Waugh
A. H. Whicher

MILVERTON.
C. Randolph

NORTH PETHERTON.

C. F. Hawkins

PILL.
T. W. S. Morgan

PORTISHEAD.
W. Monckton
C. A. Wigan

SHEPTON MALLET.
J. T. Hyatt

SOUTH PETHERTON.

S. W. MacIlwaine

TAUNTON.
Taunton Hospital
J. A. Macdonald
H. T. Rutherford

TIMSBURY.
F. Woods

WEDMORE.
J. Ford
R. P. Tyley

WELLINGTON.
J. Meredith

WELLS.

W. Fairbanks

A. L. Wade

W. A. L. Smith.

WESTON-SUPER-MARE.
H. S. Ballance
C. P. Crouch
G. B. Fraser
E. F. Martin
G. F. Rossiter
R. Roxburgh
J. W. Smith
J. Wallace
R. Wilde

WORLE.
F. St. J. Kemm

WRINGTON.
H. W. Collins

YEOVIL.
P. S. H. Colmer
W. R. M. Semple

### STAFFORDSHIRE.

STAFFORD.
J. Scott

### SURREY.

REDHILL.

C. M. Jessop

VIRGINIA WATER.
S. R. Philipps

### WARWICKSHIRE.

LEAMINGTON.
A. W. W. Hoffman

### WILTSHIRE.

BRADFORD-ON-AVON.
W. J. A. Adye
J. Beddoe

CALNE.
W. S. Batten

DEVIZES.
C. Eddowes
A. M. Gray
H. J. Mackay

FOVANT.
C. Clay

MALMESBURY.
R. Kinnier

MERE.
J. McElfatrick

PEWSEY.
H. Colman

SWINDON.

W. Howse

J. C. Maclean

WARMINSTER.
R. L. Willcox

### YORKSHIRE.

HARROGATE.
A. O. Jones.

SHEFFIELD.
S. Snell

### Scotland.

### EDINBURGHSHIRE.

EDINBURGH.
J. W. Ballantyne

### Ireland.

### ANTRIM.

BELFAST.

J. W. Browne

A. Jamison

J. A. Lindsay

### Wales.

### BRECONSHIRE.

BRECON
A. Whyte

SENNY BRIDGE.
W. R. Jones

TALGARTH.
W. Howells

### CARMARTHENSHIRE.

FERRYSIDE.
P. Williams

LLANELLY.
S. J. Roderick

### CARNARVONSHIRE.

ST. CLEARS.
R. Thomas

### GLAMORGANSHIRE.

ABERAMAN.
J. B. James

ABERDARE.
E. Jones

CAERPHILLY.
J. Lewellyn

CARDIFF.
E. T. Collins
W. T. Edwards
P. R. Griffiths
J. Milward
D. R. Paterson
J. A. Phillips
W. Price
A. Rees
A. Sheen
Medical Society
J. L. Thomas
H. R. Vachell
J. Williams

COWBRIDGE.
T. R. Hamlen-Williams

MAESTEG.
E. Davies
W. H. Thomas

MERTHYR TYDVIL.
W. W. Jones
C. E. G. Simons
J. L. W. Ward

PENARTH.
R. F. Nell

PORTH.
H. N. Davies

SWANSEA.
E. Davies
R. C. Elsworth
E. Le C. Lancaster

TREHARRIS.

W. W. Leigh

### PEMBROKESHIRE.

FISHGUARD.
H. L. Swete

HAVERFORDWEST.
E. P. Phillips

PENALLY.
B. C. Kendall

### RADNORSHIRE.

LLANDRINDOD WELLS.
A. G. Greenway

### Belgium.

COURTRAI.
Dr. Lauwers

### Italy.

GENOA.
S. Biamonti

### Switzerland.

DAVOS-PLATZ.
W. R. Huggard

### Egypt.

ALEXANDRIA.
J. Mackie

CAIRO.
M. A. Ruffer

### Cape Colony.

FORT BEAUFORT.
J. Shaw

### United States.

COLORADO.
S. E. Solly

### New Zealand.

LOWER HUTT.
J. R. Purdy

### INDEX.

Addison's disease, Supra-renal extract in, 47.
Advertiser's A B C (*Review*), 187.
Alabama, Transactions of the Medical Association of the State of (*Review*), 84.
Alcohol from a medical standpoint, The use of—J. Dacre, 10; Discussion on, 94.
Alvarenga prize, 380.
Amaurosis produced by male fern, 69.
Amblyopia, Hepatic, 67.
American Dermatological Association, Transactions of the (*Review*), 84.
American Gynecological Society, Transactions of the (*Review*), 364.
American Laryngological Association, Transactions of the (*Review*), 365.
American Ophthalmological Society, Transactions of the (*Review*), 274.
American Pediatric Society, Transactions of the (*Review*), 364.
American Physicians, Transactions of the Association of (*Review*), 272.
American Surgical Association, Transactions of the (*Review*), 273.
American year-book of medicine and surgery (*Review*), 179.
Anæmia, Bone-marrow in pernicious, 48.
Anæmia, Oxygen in the treatment of—Dr. J. M. Clarke, 232.
Anæsthesia in operations on the throat, 340.
Anæsthesia, The history of surgical —H. B. Gardner (*Review*), 362.
Annual of the universal medical sciences (*Review*), 85.
Appendicitis—G. Barling (*Review*), 182.
Appendicitis, Extra-peritoneal suppuration in—Dr. J. L. Firth, 36.
Appendicitis, Lectures on—Dr. R. T. Morris (*Review*), 258.
Appendicitis, Surgical treatment of, 161, 333.
Asepsis, Surgical—Dr. C. Beck (*Review*), 75.
Aseptic surgery—C. B. Lockwood (*Review*), 348.
Auscultatory percussion, 243.
Australasia, Intercolonial medical journal of (*Review*), 185.

Baby, The care of the—Dr. J. P. C. Griffith (*Review*), 75.
Bacillus of rheumatoid arthritis, Note on the—Dr. A. S. Wohlmann, 123; 330.
Bacteria, Pathogenic—Dr. J. McFarland (*Review*), 345.
Bacteriology, 155, 251.
Ballantyne, Dr. J. W.—The diseases and deformities of the fœtus (*Review*), 74.
Bannatyne, Dr. G. A.—Report on medicine, 330.
Barclay, W. M.—Report on surgery, 53.
Barling, G.—On appendicitis, and on perforation of gastric and duodenal ulcer (*Review*), 182.
Barnard and G. B. Stocker, W. — Medical partnerships, transfers, and assistantships (*Review*), 77.
Baron, Dr. B. J.—Report on laryngology, 338.
Baths of Great Britain, The climates and (*Review*), 361.
Beale, Drs. V. D. Harris and E. C. —The treatment of pulmonary consumption (*Review*), 257.
Beck, Dr. C.—A manual of the modern theory and technique of surgical asepsis (*Review*), 75.
Bell, J.—Notes on surgery for nurses (*Review*), 184.
Bookbinding, A short history of (*Review*), 276.
Brasher, C. W. J. — A case of ligature and division of the vasa deferentia for prostatic hypertrophy, 150.

Bread, bakehouses, and bacteria—Drs. F. J. Waldo and D. Walsh (*Review*), 81.
Bristol health report, 284.
Bristol Medico-Chirurgical Society, 91, 191, 370.
British Medical Benevolent Fund, Annual Report of the (*Review*), 367.
Brodhurst, B. E.—Observations on congenital dislocation of the hip (*Review*), 183.
Brown, Dr. A. M.—The natural arsenical waters of La Bourboule (*Review*), 267.
Browne, L.—Diphtheria and its associates (*Review*), 175.
Buboes, Treatment of, 336.

Cancer of the pharynx, 339
Cancer, Radical cure in—Dr. H. Snow (*Review*), 79.
Carbohydrates, The physiology of the. An epicriticism—Dr. F. W. Pavy (*Review*), 257.
Cardiff Medical Society, 102.
Carter, Dr. A. H.—Elements of practical medicine (*Review*), 181.
Carwardine, T.—Dermoid cyst of a branchial cleft, 145.
Cataract, Methods of operating for—G. H. Fink (*Review*), 183.
Chapman, Dr. H. C.—A manual of medical jurisprudence and toxicology (*Review*), 269.
Chemist's diary (*Review*), 86.
Children, Diets for infants and—Dr. L. Starr (*Review*), 362.
Children, Mentally-deficient — Dr. G. E. Shuttleworth (*Review*), 75.
Chloroform or ether?—J. Freeman, 137.
Cholera controversy, History of the—Sir G. Johnson (*Review*), 269.
Clarke, Dr. J. M.—On the use of oxygen in the treatment of anæmia, with a case treated by transfusion of blood, 232; Report on Medicine, 317.
Cleveland, Dr. W. F.—The prophylactic clothing of the body (*Review*), 269.
Clifton Spa, 111.
Climates and baths of Great Britain, The (*Review*), 361.
Clinical sketches (*Review*), 85.
Clinical Society of London, Transactions of the (*Review*), 275.
Clothing of the body, The prophylactic—Dr. W. F. Cleveland (*Review*), 269.
Collins, E. T.—Researches into the anatomy and pathology of the eye (*Review*), 354.
Colorado State Medical Society, Transactions of the (*Review*), 273.
Color-vision and color-blindness — Dr. J. E. Jennings (*Review*), 355.
Consumption, The treatment of pulmonary—Drs. V. D. Harris and E. C. Beale (*Review*), 257.
Counter-irritation—Dr. H. C. Gillies (*Review*), 76.
Coxa vara, 330.
Crichton-Browne, Sir J.—On dreamy mental states (*Review*), 182.
Cross, F. R.—Report on ophthalmology, 67.

Dacre, J.—The use of alcohol from a medical standpoint, 10.
Deciduoma malignum, 168.
Dermatological Society of Great Britain and Ireland, Transactions of the (*Review*), 365.
Dermatology, Bacteriology in, 251.
Dermatology, Report on—Dr. H. Waldo, 249.
Dental materia medica and therapeutics—J. Stocken (*Review*), 268.
Dermoid cyst of a branchial cleft—T. Carwardine, 145.
Devon and Exeter Medico-Chirurgical Society, 103.
Diet in sickness and in health—Mrs. E. Hart (*Review*), 179.
Diets for infants and children—Dr. L. Starr (*Review*), 362.
Digest, The medical. Appendix—Dr. R. Neale (*Review*), 181.
Diphtheria and its associates—L. Browne (*Review*), 175.
Diphtheria treated by antitoxin, 317.
Dreamy mental states — Sir J. Crichton-Browne (*Review*), 182.
Duncan, A. — Memorials of the Faculty of Physicians and Surgeons of Glasgow, 1599-1850 (*Review*), 343.
Dupouy, Dr. E.—Prostitution in antiquity (*Review*), 362.
Dutt, Dr. A. C.—Health notes for the seaside: with special reference to Whitby and district (*Review*), 267.
Dyspnœa in goitre, Sudden—C. A. Morton, 213.

Ear disease, Cerebral abscess following middle, 341.
Eczematous inflammation, 249.

Edgeworth, Dr. F. H.—Notes on a case of exophthalmic goitre, 41; The diagnosis and treatment of lumbago, 304.
Edinburgh hospital reports (*Review*), 82.
Edinburgh Obstetrical Society, Transactions of the (*Review*), 83.
Editorial note, 1.
Ehlers, Dr. E. H.—Conditions under which leprosy has declined in Iceland (*Review*), 263.
Electricity, Medical—Dr. H. L. Jones (*Review*), 80.
English illustrated magazine (*Review*), 87.
Epidemics, The prevention of—F. Vacher (*Review*), 268.
Epidemiological Society of London, Transactions of the (*Review*), 366.
Épilepsie, l'hystérie et l'idiotie, Recherches cliniques et thérapeutiques sur l' (*Review*), 272.
Erysipelas, Anti-streptococcus serum in, 327.
Ether? Chloroform or—J. Freeman, 137.
Exophthalmic goitre—Dr. F. H. Edgeworth, 41.
Eye, Anatomy and pathology of the—E. T. Collins (*Review*) 354.
Eye, Diseases of the—Dr. H. R. Swanzy (*Review*), 80.
Eye in its relation to health, The—Dr. C. Prentice (*Review*), 363.
Eyesight and school life—S. Snell (*Review*), 356.

Fernie, Dr. W. T.—Herbal simples (*Review*), 77.
Fink, G. H.—Methods of operating for cataract (*Review*), 183.
Finlayson, Dr. J.—Galen (*Review*), 265.
Firth, Dr. J. L.—Extra-peritoneal suppuration in perforating appendicitis, 36.
Fisher, Dr. T.—Report on medicine, 47.
Fœtus, The diseases and deformities of the—Dr. J. W. Ballantyne (*Review*), 74.
Food and drugs, Analysis of—T. H. Pearmain and C. G. Moor (*Review*), 262.
Fox, Dr. B. B.—A case of hystero-epilepsy, 224.
Freeman, J.—Chloroform or ether?; 137.
Fürbringer, Dr. P.—Text-book on diseases of the kidneys and urinary organs (*Review*), 71.

Galen—Dr. J. Finlayson (*Review*), 265.
Gardner, H. B.—The history of surgical anæsthesia (*Review*), 362.
Gasserian ganglion, Operations on the, 54.
Gillies, Dr. H. C.—The theory and practice of counter-irritation (*Review*), 76.
Gimlette, J. D.—Myxœdema and the thyroid gland (*Review*), 78.
Glasgow, Memorials of the Faculty of Physicians and Surgeons of, 1599–1850—A. Duncan (*Review*), 343.
Goitre, Exophthalmic—Dr. F. H. Edgeworth, 41.
Goitre, Sudden dyspnœa in—C. A. Morton, 213.
Government services, Regulations as to defects which disqualify for admission into the—N. C. Macnamara (*Review*), 183.
Grandin and G. W. Jarman, Drs. E. H.—Pregnancy, labor, and the puerperal state (*Review*), 259.
Grant College Medical Society, Transactions of the (*Review*), 185.
Great Britain, The climates and baths of (*Review*), 361.
Greene, R.—The Schott treatment for chronic heart diseases (*Review*), 266.
Griffith, Dr. J. P. C.—The care of the baby (*Review*), 75.
Guy's hospital reports (*Review*), 184.

Hand, The clinical significance of the human—Dr. A. S. Wohlmann, 117.
Hansen and C. Looft, Drs. G. A.—Leprosy (*Review*), 73.
Harris and E. C. Beale, Drs. V. D.—The treatment of pulmonary consumption (*Review*), 257.
Harrison, R.—Some points in the practice of lithotrity or litholapaxy (*Review*), 79.
Harsant, W. H.—Report on otology, 341; Report on surgery, 245.
Hart, E.—The truth about vaccination (*Review*), 81.
Hart, Mrs. E.—Diet in sickness and in health (*Review*), 179.
Health notes for the seaside—Dr. A. C. Dutt (*Review*), 267.
Heart-disease, Exercises in, 240.
Heart diseases, The Schott treatment for chronic—R. Greene (*Review*), 266.
Heart, Relation of the, to pregnancy, 51.

Heart, Syphilis of the, 50.
Herbal simples—Dr. W. T. Fernie (*Review*), 77.
Hernia, The operation for strangulated—C. A. Morton, 23.
Hip, Congenital dislocation of the —B. E. Brodhurst (*Review*), 183.
Hip-joint, Diseases of the, 330.
Hospital annual, Burdett's (*Review*), 276.
Hutchinson, J.—A smaller atlas of illustrations of clinical surgery (*Review*), 178.
Hyde and F. H. Montgomery, Drs. J. N.—A manual of syphilis and the venereal diseases (*Review*), 357.
Hygiene and public health—Dr. L. C. Parkes (*Review*), 80.
Hystero-epilepsy, A case of—Dr. B. B. Fox, 224.

'Ιατρική 'Επιθεώρησις (*Review*), 185.
Impey, Dr. S. P.—A handbook on leprosy (*Review*), 360; Leprosy in South Africa. On spontaneous recovery from leprosy (*Review*), 263.
Index-catalogue of Library of the Surgeon-General's Office, United States Army (*Review*), 266.
Iowa State Medical Society, Transactions of the (*Review*), 84.
Ireland, Transactions of the Royal Academy of Medicine in (*Review*), 364.

Jarman, Drs. E. H. Grandin and G. W.—Pregnancy, labor, and the puerperal state (*Review*), 259.
Jenner, 255.
Jennings, Dr. J. E.—Color-vision and color-blindness (*Review*), 355.
Johns Hopkins hospital reports (*Reviews*), 81, 269.
Johnson, Sir G.—History of the cholera controversy (*Review*), 269.
Joints and spine, Diseases of the—H. Marsh (*Review*), 72.
Jones, Dr. H. L.—Medical electricity (*Review*), 80.
Jurisprudence and toxicology, Medical—Dr. H. C. Chapman (*Review*), 269.

Keen and J. W. White, Drs. W. W. [Eds.]—An American text-book of surgery (*Review*), 261.
Kenwood, Dr. H. R.—Public health laboratory work (*Review*), 268.
King's College hospital reports (*Reviews*), 184, 363.

La Bourboule, The natural arsenical waters of—Dr. A. M. Brown (*Review*), 267.
Langerhans, Dr. R.—Grundriss der pathologischen anatomie (*Review*), 344.
Laryngology, Report on—Dr. B. J. Baron, 338.
Laryngoscope, The (*Review*), 366.
Larynx, Malignant disease of the, 339.
Lawrence, Dr. A. E. A.—The evils of marriage and pregnancy in women who do not possess sound pelvic organs, 289.
Leprosy, A handbook on (*Review*), 360; Leprosy in South Africa—Dr. S. P. Impey (*Review*), 263.
Leprosy—Drs. G. A. Hansen and C. Looft (*Review*), 73.
Leprosy in Iceland—Dr. E. H. Ehlers (*Review*), 263.
Leprosy in the British Islands—Dr. G. Newman (*Review*), 173.
Leprosy, On spontaneous recovery from—Dr. S. P. Impey (*Review*), 263.
Letts's diaries (*Review*), 366.
Leucocythæmia, 158.
Lewis, Dr. P. G.—Early scoliosis, or curable curvatures of the spine (*Review*), 79.
Library of the Bristol Medico-Chirurgical Society, 104, 206, 281, 373.
Library of the Surgeon-General's Office, United States Army, Index-catalogue of (*Review*), 266.
Lindsay, Dr. R.—An essay on malaria and its consequences (*Review*), 181.
Lips, Disorders of the vermilion portion of the, 250.
Lithotrity, Some points in the practice of—R. Harrison (*Review*), 79.
Local medical notes, 110, 209, 284, 378.
Lockwood, C. B.—Aseptic surgery (*Review*), 348; Traumatic infection (*Review*), 259.
Lockwood, Dr. G. R.—A manual of the practice of medicine (*Review*), 257.
London Temperance Hospital report (*Review*), 363.
London, Transactions of the Clinical Society of (*Review*), 275.
London, Transactions of the Medical Society of (*Review*), 275.

London, Transactions of the Obstetrical Society of (*Review*), 82.
Looft, Drs. G. A. Hansen and C.—Leprosy (*Review*), 73.
Lumbago, The diagnosis snd treatment of—Dr. F. H. Edgeworth, 304.
Lupus erythematosus, 254.
Lymphadenoma, 158.

McFarland, Dr. J.—A text-book upon the pathogenic bacteria (*Review*), 345.
Macnamara, N. C.—Regulations as to defects which disqualify for admission into the government services (*Review*), 183.
Malaria, 156; Dr. R. Lindsay (*Review*), 181.
Manual for the church lads' brigade medical staff corps (*Review*), 80.
Marriage and pregnancy, On some evils of—Dr. A. E. A. Lawrence, 289.
Marsh, H.—Diseases of the joints and spine (*Review*), 72.
Martin, Dr. S.—Functional and organic diseases of the stomach (*Review*), 176.
Materia medica and therapeutics—Dr. J. V. Shoemaker (*Review*), 267.
Medical annual (*Review*), 185.
Medical diary (*Review*), 86.
Medicine, Bacteriology in, 155.
Medicine, Elements of practical—Dr. A. H. Carter (*Review*), 181.
Medicine, Practice of—Dr. G. R. Lockwood (*Review*), 257.
Medicine, Reports on—Dr. J. M. Clarke, 317; Dr. T. Fisher, 47; Dr. G. Parker, 155; Dr. R. S. Smith, 240.
Medico - Chirurgical Transactions (*Review*), 184.
Michigan State Medical Society Transactions of the (*Review*), 84.
Middlesex hospital reports (*Review*), 270.
Monro, Dr. T. K.—A history of the chronic degenerative diseases of the central nervous system (*Review*), 265.
Montgomery, Drs. J. N. Hyde and F. H.—A manual of syphilis and the venereal diseases (*Review*), 357.
Moor, T. H. Pearmain and C. G.—Aids to the analysis of food and drugs (*Review*), 262.
Morris, H.—Injuries and diseseas of the genital and urinary organs (*Review*), 178.
Morris, Dr. R. T.—Lectures on appendicitis and notes on other subjects (*Review*), 258.
Morton, C. A—Report on surgery, 161; The causation and treatment of sudden dyspnœa in goitre, 213; Two years' experience of the operation for strangulated hernia at the Bristol General Hospital, 23.
Murray, Dr. W.—Rough notes on remedies (*Review*), 267.
Mydriatic, A, 70.
Myxœdema and the thyroid gland—J. D. Gimlette (*Review*), 78.

Nasal obstruction, Chronic, 338.
Neale, Dr. R.—The medical digest. Appendix (*Review*), 181.
Nervous diseases, History of chronic—Dr. T. K. Monro (*Review*), 265.
Neurectomy, 54.
Neurologie & d'hypnologie, Journal de (*Review*), 86.
New England States, Vital statistics of the (*Review*), 81.
New Hampshire Medical Society, Transactions of the (*Review*), 274.
New Jersey, Transactions of the Medical Society of (*Review*), 84.
Newman, Dr. G.—On the history of the decline and final extinction of leprosy as an endemic disease in the British Islands (*Review*), 173.
Newport Medical Society, 373.
New York Academy of Medicine, Transactions of the (*Review*), 82.
New York, Transactions of the Medical Society of the State of (*Review*), 84.
Nocard, E.—The animal tuberculoses, and their relation to animal tuberculosis (*Review*), 78.
Norris, Dr. R. C. [Ed.]—An American text-book of obstetrics (*Review*), 348.
Nurses' diary (*Review*), 86.
Nurses, Surgery for—J. Bell (*Review*), 184.

Obstetrical Society of London, Transactions of the (*Review*), 82.
Obstetrics, An American text-book of—Dr. R. C. Norris [Ed.] (*Review*), 348.
Obstetrics, Report on—Dr. W. C. Swayne, 168.
Ohio State Medical Society, Transactions of the (*Review*), 84.
Ophthalmia, Epidemic—Dr. S. Stephenson (*Review*), 356.

Ophthalmological Society of the United Kingdom, Transactions of the (*Review*), 365.
Ophthalmology, Report on—F. R. Cross, 67.
Otology, Report on—W. H. Harsant, 341.
Ovaries and Fallopian tubes, Surgical diseases of the—J. B. Sutton (*Review*), 353.
Oxaluria, 161.
Oxygen in the treatment of anæmia —Dr. J. M. Clarke, 232.

Papillomata affecting both vocal cords, 338.
Parke, Davis & Co., Catalogue of (*Review*), 276.
Parker, Dr. G.—Report on medicine, 155.
Parkes, Dr. L. C.—Hygiene and public health (*Review*), 80.
Partnerships, Medical—W. Barnard and G. B. Stocker (*Review*), 77.
Pasteur, Velada fúnebre en honra del eminente sabio francés Luis (*Review*), 265.
Pathologischen anatomie, Grundriss der—Dr. R. Langerhans (*Review*), 344.
Pavy, Dr. F. W.—The physiology of the carbohydrates. An epicriticism (*Review*), 257.
Pearmain and C. G. Moor, T. H. —Aids to the analysis of food and drugs (*Review*), 262.
Pediatrics (*Review*), 86.
Pharmacopœia for diseases of the skin—J. Startin [Ed.] (*Review*), 268.
Pharmacopœia of the Evelina Hospital for Sick Children, The (*Review*), 268.
Pharynx, Cancer of the, 339.
Philadelphia, Transactions of the College of Physicians of (*Review*), 273.
Physiology, A manual of—Dr. G. N. Stewart (*Review*), 256.
Plymouth Medical Society, 201.
Poisoning, Notes on self-—Dr. C. J. Whitby, 307.
Pompeian surgery and surgical instruments, 114.
Pountney, Dr. W. E.—A case of needle in foot revealed by the X rays, 238.
Pregnancy, labor, and the puerperal state—Drs. E. H. Grandin and G. W. Jarman (*Review*), 259.
Pregnancy, The heart in relation to, 51.
Premature labour, Induced—Dr. J. G. Swayne, 2, 126.
Prentice, Dr. C.—The eye in its relation to health (*Review*), 363.
Prichard, A.—A few medical and surgical reminiscences (*Review*), 180.
Prostatic hypertrophy, Ligature and division of the vasa deferentia for —C. W. J. Brasher, 150.
Prostitution in antiquity—Dr. E. Dupouy (*Review*), 362.
Public health laboratory work—Dr. H. R. Kenwood (*Review*), 268.
Puerperal fever, Anti-streptococcus serum in, 328.
Purdy, Dr. C. W.—Practical uranalysis and urinary diagnosis (*Review*), 267.

Retinitis, Albuminuric 68.
Remedies, Rough notes on—Dr. W. Murray (*Review*), 267.
Reminiscences—A. Prichard (*Review*), 180.
Rheumatoid arthritis, Note on the bacillus of—Dr. A. S. Wohlmann, 123; 330.
Ringworm, 252.
Roberts, C.—The medical inspection of, and physical education in, schools (*Review*), 363.

Saint Bartholomew's hospital reports (*Review*), 270.
Scarlet fever, Diphtheria antitoxin in, 324; Anti-streptococcus serum in, 325.
School life, Eyesight and—S. Snell (*Review*), 356.
Schools, The medical inspection of, and physical education in—C. Roberts (*Review*), 363.
Schott treatment for chronic heart diseases, The—R. Greene (*Review*), 266.
Scoliosis, Early—Dr. P. G. Lewis (*Review*), 79.
Scraps, 113, 211, 287, 380.
Senn, N.—Principles of surgery (*Review*), 177.
Septicæmia, Anti-streptococcus serum in acute, 329.
Serumtherapy, 49, 317.
Shoemaker, Dr. J. V.—A practical treatise on materia medica and therapeutics (*Review*), 267.
Shuttleworth, Dr. G. E.—Mentally-deficient children (*Review*), 75.

Sick, Preparations for the, 87, 187, 277, 368.
Skiagraphy, Archives of clinical (*Review*), 186.
Skin, The histopathology of the diseases of the—Dr. P. G. Unna (*Review*), 357.
Smith, Dr. R. S.—A case of double spasmodic torticollis, 297; Report on medicine, 240.
Snell, S.—Eyesight and school life (*Review*), 356.
Snow, Dr. H.—The conditions of radical cure in cancer (*Review*), 79.
Societies: Bristol Medico-Chirurgical, 91, 191, 370; Cardiff Medical, 102; Devon and Exeter Medico-Chirurgical, 103; Newport Medical, 373; Plymouth Medical, 201; Swansea Medical, 203; Torquay Medical, 104, 205.
Spinal cord disease in relation to spinal blood-vessels—Dr. R. T. Williamson (*Review*), 78.
Spine, Diseases of the joints and —H. Marsh (*Review*), 72.
St. Thomas's hospital reports (*Review*), 271.
Starr, Dr. L.—Diets for infants and children in health and disease (*Review*), 362.
Startin, J. [Ed.]—A pharmacopœia for diseases of the skin (*Review*), 268.
Stephenson, Dr. S.—Epidemic ophthalmia (*Review*), 356.
Stewart, Dr. G. N.—A manual of physiology (*Review*), 256.
Stocken, J.—Dental materia medica and therapeutics (*Review*), 268.
Stocker, W. Barnard and G. B. — Medical partnerships, transfers, and assistantships (*Review*), 77.
Stomach, Diseases of the—Dr. S. Martin (*Review*), 176.
Stomach, Perforating ulcer of the, 245.
Surgery, An American text-book of —Drs. W. W. Keen and J. W. White [Eds.] (*Review*), 261.
Surgery for nurses—J. Bell (*Review*), 184.
Surgery, Illustrations of clinical—J. Hutchinson (*Review*), 178.
Surgery, Principles of—N. Senn (*Review*), 177.
Surgery, Reports on — W. M. Barclay, 53; W. H. Harsant, 245; C. A. Morton, 161; Dr. J. Swain, 330.
Sutton, J. B.—Surgical diseases of the ovaries and Fallopian tubes, including tubal pregnancy (*Review*), 353.
Swain, Dr. J.—Report on surgery, 330.
Swansea Medical Society, 203.
Swanzy, Dr. H. R.—A handbook of the diseases of the eye and their treatment (*Review*), 80.
Swayne, Dr. J. G.—Induced premature labour in certain diseases of the mother not obstructing delivery, 2, 126.
Swayne, Dr. W. C.—Report on obstetrics, 168.
Syphilis and the venereal diseases, A manual of—Drs. J. N. Hyde and F. H. Montgomery (*Review*), 357.
Syphilis of the heart, 50.

Tetanus treated by antitoxin, 49.
Texas State Medical Association, Transactions of the (*Review*), 84.
Tic douloureux, Surgical treatment of, 53.
Tonsillitis, Anti-streptococcus serum in membranous, 327.
Torticollis, On double spasmodic—Dr. R. S. Smith, 297.
Torquay Medical Society, 104, 205.
Toxicology, Medical jurisprudence and—Dr. H. C. Chapman (*Review*), 269.
Tracheal (Intra-) injections, 339.
Traumatic infection—C. B. Lockwood (*Review*), 259.
Tsetse fly-disease, 156.
Tuberculoses, The animal — E. Nocard (*Review*), 78.
Typhoid fever, 158.

Unna, Dr. P. G.—The histopathology of the diseases of the skin (*Review*), 357.
Urinary diagnosis, Practical uranalysis and—Dr. C. W. Purdy (*Review*), 267.
Urinary organs, Diseases of the kidneys and—Dr. P. Fürbringer (*Reviem*), 71.
Urinary organs, Injuries and diseases of the genital and—H. Morris (*Review*), 178.

Vaccination, Note on, 160.
Vaccination, The truth about—E. Hart (*Review*), 81.

Vacner, F.—The prevention of epidemics (*Review*), 268.
Virginia, Transactions of the Medical Society of (*Review*), 274.
Visiting list (*Review*), 367.
Vitreous with synchysis, Disorganisation of the, 69.

Waldo and D. Walsh, Drs. F. J.—Bread, bakehouses, and bacteria (*Review*), 81.
Waldo, Dr. H.—Report on dermatology, 249.
Walsh, Drs. F. J. Waldo and D.—Bread, bakehouses, and bacteria (*Review*), 81.
Weitbrecht, Retinacula of, 93.
West London medical journal (*Review*), 86.
West London Medico-Chirurgical Society, Proceedings of the (*Review*), 82.
Whitby, Dr. C. J.—Notes on self-poisoning and its treatment, with special reference to some physical methods, 307.
White, Drs. W W. Keen and J. W. [Eds.]—An American text-book of surgery (*Review*), 261.
Williamson, Dr. R. T.—On the relation of diseases of the spinal cord to the distribution and lesions of the spinal blood-vessels (*Review*), 78.
Wohlmann, Dr. A. S.—The clinical significance of the human hand, 117; Note on the bacillus of rheumatoid arthritis, 123.

X rays, 112, 238.

Year-book of scientific societies (*Review*), 276.
Year-book of treatment (*Review*), 85.

---

J. W. Arrowsmith, Printer, Quay Street, Bristol.

